# Simultaneous Force Regression and Movement Classification of Fingers *via* Surface EMG within a Unified Bayesian Framework

**DOI:** 10.3389/fbioe.2018.00013

**Published:** 2018-02-26

**Authors:** Tara Baldacchino, William R. Jacobs, Sean R. Anderson, Keith Worden, Jennifer Rowson

**Affiliations:** ^1^Department of Automatic Control and Systems Engineering, University of Sheffield, Sheffield, United Kingdom; ^2^Dynamics Research Group, Department of Mechanical Engineering, University of Sheffield, Sheffield, United Kingdom; ^3^Insigneo Institute for in silico Medicine, University of Sheffield, Sheffield, United Kingdom

**Keywords:** sEMG signals, finger force regression, finger movement classification, variational Bayes, multivariate mixture of experts, prosthetic hand

## Abstract

This contribution presents a novel methodology for myolectric-based control using surface electromyographic (sEMG) signals recorded during finger movements. A multivariate Bayesian mixture of experts (MoE) model is introduced which provides a powerful method for modeling force regression at the fingertips, while also performing finger movement classification as a by-product of the modeling algorithm. Bayesian inference of the model allows uncertainties to be naturally incorporated into the model structure. This method is tested using data from the publicly released NinaPro database which consists of sEMG recordings for 6 degree-of-freedom force activations for 40 intact subjects. The results demonstrate that the MoE model achieves similar performance compared to the benchmark set by the authors of NinaPro for finger force regression. Additionally, inherent to the Bayesian framework is the inclusion of uncertainty in the model parameters, naturally providing confidence bounds on the force regression predictions. Furthermore, the integrated clustering step allows a detailed investigation into classification of the finger movements, without incurring any extra computational effort. Subsequently, a systematic approach to assessing the importance of the number of electrodes needed for accurate control is performed *via* sensitivity analysis techniques. A slight degradation in regression performance is observed for a reduced number of electrodes, while classification performance is unaffected.

## 1 Introduction

Estimation of user intention from surface electromyographic (sEMG) signals is a key challenge in the design of active prosthetics, and this method of control has been a thriving area of research for a few decades (Saridis and Gootee, 1982). The harnessing of sEMG signals allows the user to actively control the device, while providing a relatively simple, non-invasive and low cost human-machine-interface. Highly dexterous active arm prosthetics will enable amputees to perform tasks which are not possible with the current state-of-the-art. However, achieving refined control of upper limb prosthetics is particularly challenging due to the very high number of degrees-of-freedom (DoF) present in the extremities of the arm.

In order to achieve highly dexterous control of prosthetic arms, control schemes are used that combine classification of movement-type with regression for proportional force control (Castellini and van der Smagt, 2009). Classification of hand/wrist movements (including grasping) has been tackled successfully (Ferguson and Dunlop, 2002; Farrell and Weir, 2008; Atzori et al., 2012). Performing finger movement classification is a more difficult task: first, sEMG signals are smaller in amplitude for finger movements, and second, muscles responsible for finger activations lie deep beneath the skin surface and so the signals recorded at the skin are subject to nonlinear transformations by forearm tissues (Al-Timemy et al., 2013). Several authors have tackled single and multiple finger movement classification using a variety of combinations of sEMG feature extractions (FE) and classifiers, with varying degrees of success (Jiang et al., 2005; Castellini and van der Smagt, 2009; Naik et al., 2009; Tenore et al., 2009; Kanitz et al., 2011; Al-Timemy et al., 2013; Kumar et al., 2013; Li et al., 2015; Naik and Nguyen, 2015; Jarrassé et al., 2017) (refer to Table 1 for a comprehensive list).

**Table 1:** A table of related work found in the literature for both finger movement classification and force regression.

# electrodes	Feature Extraction	Classifier (regressor)	Finger moves	# of subjects	Accuracy	Reference
4	WT	ANN	6 s	10H	>80%	Jiang et al. (2005)
4	TD	ANN	4 m	7H	96%	Naik et al. (2009)
32, 19	TD	MLP	10 s	5H, 1A	90%	Tenore et al. (2009)
10	–	SVM	4 m	12H	89.7%	Castellini and van der Smagt (2009)
10	–	(SVM)	4 m	12H	7.98% (NRMSE)	Castellini and van der Smagt (2009)
12	2–3 TDs	SVM	12 s	5H, 1A	>80%	Kanitz et al. (2011)
9	Filtering	(SVM)	6 s, 5 m	12H	<8% (NRMSE)	Castellini and Kõiva (2012)
2	WT	SVM	4 s	11H, 1A	93%	Kumar et al. (2013)
12, 11	TDAR	LDA	11 s, 4 m	10H, 6A	>95%	Al-Timemy et al. (2013)
12	mDWT	(NK)	6 s, 3 m	40H	91.74% (R2)	Gijsberts et al. (2014)
6	TDAR	ANN	5 s	5H	89.4%	Li et al. (2015)
6	TDAR	(Quadratic)	5 s	5H	0.15 (MAE)	Li et al. (2015)
2	TDAR	ANN	5 s, 5 m	8H	92%	Naik and Nguyen (2015)
12 pairs	Various	LDA	5 s	3A	<83%	Jarrassé et al. (2017)

*“# electrodes” gives the number of electrodes for healthy, amputee. The type of feature extraction used is given in the second column: WT (wavelet transform), TD (time domain), TDAR (time domain autoregressive), and mDWT (marginal discrete wavelet transform). The classifiers/regressors are given by: ANN (artificial neural network), MLP (multilayer perceptron), SVM (support vector machine), LDA (linear discriminant analysis), and NK (nonlinear kernel). In “Finger Moves” column, “s” = single and “m” = multiple. H and A in the “# of subjects” column correspond to healthy and amputee subjects, respectively. Classifier accuracy is the percentage of correct predictions, and regressor performance uses NRMSE (normalized root mean square error), *R*^2^, or MAE (mean absolute error)*.

Recently, regression between the user’s intent, *via* sEMG signals, and the force applied at the fingertips has been performed (Castellini and van der Smagt, 2009; Castellini and Kõiva, 2012; Gijsberts et al., 2014; Li et al., 2015) (refer to Table 1 for references). Regression for proportional force control is a natural and accurate form of control since sEMG signals are related to the force a muscle is applying (Luca, 1997). The whole control scheme, consisting of a classifier coupled with a regressor, is hence capable of predicting the movement being performed along with how much force the subject is exerting.

Movement dynamics of a patient observed *via* sEMG can vary substantially over time due to, e.g., the fatiguing of muscle, motor unit noise, and electrode movement (Luca, 1997). Due to this variability, dynamic models of movement would be improved by intrinsic characterization of uncertainty within the modeling framework to aid robust control design. To date, this aspect of user intention estimation has generally been neglected. In order to address this gap, the authors propose a new method for classifying movement-type and regressing force based on a mixture of experts (MoE) model, identified within a Bayesian framework which intrinsically characterizes model uncertainty. Further advantages of this approach are: (i) the method simultaneously performs classification and regression, which simplifies the model representation and estimation process, (ii) the model can represent both linear and nonlinear dynamics, and (iii) the estimation algorithm consists of an iterative sequence of closed form expressions, which are computationally simple to execute.

The MoE model used in this work probabilistically divides the input space of a system by means of *gates*, while individual regression *experts* specialize on certain regions of the input space. Thus the adoption of a MoE model exploits the linear relationship between the sEMG signals and the force for each finger movement, giving rise to a more natural modeling description. Additionally, classification is also performed since it is a by-product of the MoE structure which is integral to the model. Thus force regression and movement classification of fingers are implemented within a single unified framework.

Bayesian inference naturally incorporates uncertainty into the training of the model by specifying distributions over the parameters. A useful by-product is the evaluation of confidence bounds of model predictions, hence providing a natural check on the model’s predictive capabilities. Due to the Bayesian setting, overfitting is avoided and a metric naturally arises for choosing the appropriate number of experts. Bayesian inference also opens up the possibility of performing patient-specific modeling by incorporating patient-specific heuristics to guide the model building process in order to provide much more accurate predictions for clinical applications. One such approach, for example, is to instruct the Bayesian model averaging process in clinical applications, which has been successfully applied to sepsis and heart failure clinical datasets (Visweswaran et al., 2010).

Cost and power consumption are predominant factors for the success of a method for clinical application, and they can be reduced by minimizing the number of electrodes used for classification and regression. In this work, this problem is addressed by applying sensitivity analysis (SA) techniques in order to provide a methodical framework for assessing the importance of the individual and grouped sEMG signals on the output force DoF. SA allows an increased understanding of the relationships between the input and output variables of a system/model and has been used extensively within the biomechanics literature, see for example, Batterbee et al. (2011) and Becker et al. (2011).

In this study, the authors build on previous analytical and computational work (Baldacchino et al., 2016) and demonstrate that the proposed multivariate Bayesian MoE model is a suitable candidate for finger force regression and finger classification using sEMG signals since accurate performance, comparable to that found in the literature, was achieved for both regression and classification. The novel work presented in this article provides a powerful method for actively controlling highly dexterous prosthetic and robotic hands for rehabilitation purposes.

The rest of the article is structured as follows. Section 2 describes the sEMG/force dataset used in this article, along with preprocessing methods for the sEMG signals. This section also summarizes the main analysis approaches. The multivariate MoE model is introduced in Section 2.2, and the Bayesian parameter update equations are given in Section 2.3. Section 2.6 gives details of the SA framework used in this work. The results are presented collectively in Section 3. An in-depth discussion regarding the results obtained is given in Section 4. Finally, conclusions on the work presented are drawn in Section 5.

## 2 The Data and the Methodology

### 2.1 NinaPro Data

The dataset analyzed in this article is comprised of the second version of the publicly released NinaPro (Non-invasive Adaptive Prosthetics) database (Atzori et al., 2012; Gijsberts et al., 2014). The NinaPro database contains sEMG recordings collected from 40 intact subjects while performing a large number of common hand/finger movements and grasping actions. Of particular interest is the dataset containing measurements of the sEMG and the corresponding applied forces at the fingertips for different finger and thumb movements.

**Figure 1:**
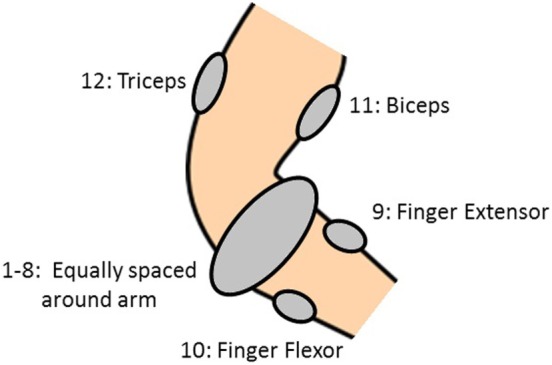
Location of the 12 sEMG electrodes on the arm.

Each subject had 12 electrodes attached to their forearm and upper arm in order to measure sEMG signals. The 12 electrodes were placed in such a way that some electrodes provided dense sampling while others targeted specific muscles. Eight electrodes (1–8) were equally spaced around the forearm at the height of the radio-humeral joint, one electrode each was placed on the finger extensor (9) and flexor (10) muscles, and on the biceps (11) and triceps (12), see Figure 1. Six DoF force measurements were considered: flexion of the five digits (little, ring, middle, index and thumb) along with abduction of the thumb. These force signals were collected by way of a Finger-Force Linear Sensor (FFLS) (Kõiva et al., 2012). Each subject was asked to produce a set of nine force patterns, given in Table 2, by pressing down with one or more digits of the dominant hand in response to an external stimulus. Each force pattern was repeated six times, and a rest period was enforced in between each movement and each repetition in order to prevent muscle fatigue. More information and detail regarding the setup and data collection can be found in Gijsberts et al. (2014), Nin (2014).

**Table 2:** Description of the 9 force patterns.

#	Movement description
F1–F4	Flexion of little through to index fingers.
F5/F6	Abduction/Flexion of the thumb.
F7	Flexion of the index and little finger.
F8	Flexion of the ring and middle finger.
F9	Flexion of the index finger and the thumb.

The data preprocessing steps employed in this article follow the steps proposed in Gijsberts et al. (2014), since a benchmark for force regression on the NinaPro data has been set. The data was first split into a training and testing set based on the repetition of movements; the second and fifth repetition for each movement were used for testing and the four remaining repetitions were used for training. All the data were then standardized to be zero mean and unit variance, using statistics calculated solely from the training set.

A common practice used in smoothing sEMG signals is to first segment the signal into overlapping windows, and features are then extracted from each window. The sEMG signals were sampled at 2,000 Hz, and following on from Gijsberts et al. (2014) windows of 400 ms (800 samples) with a sliding window increment of 10 ms (20 samples) were used. For computational feasibility the training set was subsampled by a factor of 10 (at regular intervals) resulting in approximately 3,000 data points for training. The subsample rate used here is slightly higher than that used by Gijsberts et al. (2014) for regression analysis but preliminary analysis indicated that a higher subsample rate achieved a better performance. The MoE model is trained using the extracted sEMG features as inputs to the modeling algorithm.

Preliminary results using multivariate MoE for force regression alluded to low-dimensional FE representations outperforming high dimensional FEs (Baldacchino et al., 2015). Thus commonly used low dimensional time-domain FEs for sEMG signals are considered in this work, see Table 3. All the FEs considered have dimensionality equal to the number of sEMG channels (in this case 12). All the FEs, except for FILT, were performed on the windowed data as outlined in the previous paragraph. For FILT FE, a zero-phase second-order low pass Butterworth filter with a cutoff frequency of 2 Hz was applied to the full wave rectified sEMG data, separately for the training and test datasets. In order to obtain a similar number of data points to the other FE datasets, this dataset was subsampled at regular intervals of 200 samples.

**Table 3:** A table showing the different FEs, used on each channel of the sEMG data, analyzed in this article.

Feature extraction (FE)	Definition (per channel)	References
Mean absolute value (MAV)	$\text{x}=\frac{\text{1}}{{\text{N}}_{\text{w}}}\sum_{\text{n}=\text{1}}^{{\text{N}}_{\text{w}}}\;|{\tilde{\text{x}}}_{\text{n}}|$	Hudgins et al. (1993), Hargrove et al. (2008), Artemiadis and Kyriakopoulos (2011), and Kuzborskij et al. (2012)
Waveform length (WL)	$\text{x}=\sum_{\text{n}=\text{2}}^{{\text{N}}_{\text{w}}}\;|{\tilde{\text{x}}}_{\text{n}}-{\tilde{\text{x}}}_{\text{n}-\text{1}}|$	Hudgins et al. (1993), Hargrove et al. (2008), Artemiadis and Kyriakopoulos (2011), and Kuzborskij et al. (2012)
Root mean square (RMS)	$\text{x}={\left(\frac{\text{1}}{{\text{N}}_{\text{w}}}\sum_{\text{n}=\text{1}}^{{\text{N}}_{\text{w}}}\;{\tilde{\text{x}}}_{\text{n}}^{\text{2}}\right)}^{\frac{\text{1}}{\text{2}}}$	Gijsberts et al. (2014) and Hahne et al. (2014)
Butterworth filter (FILT)	See text	Atzori et al. (2012) and Lenzi et al. (2012)

*The extracted features are denoted by *x* which are computed from the windowed sEMG signal *x*˜ of length ${\text{N}}_{\text{w}}$. The “References” column provides references from the biomedical, robotics and exoskeletons literature where the different types of FEs have been implemented*.

### 2.2 Multivariate Bayesian Mixture of Experts

The only instance of an MoE model applied to myoprosthetic control appears in Hahne et al. (2014) for regression analysis of wrist movements. The model was limited to two experts and trained using iterative reweighted least squares separately for each DoF. In Section 2.2.1, a sophisticated MoE is defined which is capable of accommodating unlimited DoFs and achieving analytical update solutions. The MoE model structure used in this article follows the work of Ueda and Ghahramani (2002), and further developed in Baldacchino et al. (2016). This particular choice of MoE model was adopted for two reasons; tractable closed form update equations for the parameters are obtained, and it is easily extended to accommodate multivariate outputs. These two characteristics reduce the computational effort during the training phase resulting in fast training times. The model used in this article is thus referred to as the multivariate Bayesian MoE model. Since a Bayesian approach to training the model is employed, prior distributions for the random variables need to be specified and these are given in Section 2.2.2.

#### 2.2.1 Multivariate MoE Regression Model

The *d^y^* DoF force signal, ${\text{y}}_{n}=\left[y_{n}^{1},\ldots ,y_{n}^{d^{y}}\right]$, at time instant *n*, can be represented by a MoE model with *M* regression experts, given by

(1) $${\text{y}}_{n}=\overset{M}{\underset{i=1}{\sum }}\;g_{i}\;\left({\text{x}}_{n},\theta_{i}^{g}\right)\;f_{i}\left({\text{x}}_{n},W_{i}\right),$$

where ${\text{x}}_{n}=\left[x_{n}^{1},\ldots ,x_{n}^{d^{x}}\right]$ is the *d^x^* dimensional input vector consisting of the extracted sEMG features. The *i*th gating function, *g_i_*(⋅), is a *normalized Gaussian* function, given by

(2) $$g_{i}\left({\text{x}}_{n},\pi_{i},\theta_{i}^{g}\right)=\frac{\pi_{i}N\;\left({\text{x}}_{n}|\mu_{i},\Lambda_{i}^{-1}\right)}{\sum_{l=1}^{M}\;\pi_{l}N\;\left({\text{x}}_{n}|\mu_{l},\Lambda_{l}^{-1}\right)},$$

where $\theta_{i}^{g}=\left[\mu_{i},\Lambda_{i}\right]$; $\mu ={\left\{\mu_{i}\right\}}_{i=1}^{M}$ is the mean and $\Lambda^{-1}={\left\{\Lambda_{i}^{-1}\right\}}_{i=1}^{M}$ is the covariance matrix. $\pi ={\left\{\pi_{i}\right\}}_{i=1}^{M}$ are the mixing coefficients satisfying π_i_ ≥ 0 and $\sum_{i=1}^{M}\;\pi_{i}=1$. $g_{i}\left({\text{x}}_{n},\pi_{i},\theta_{i}^{g}\right)$ is the posterior conditional probability that ***x****_n_* is assigned to the partition corresponding to the *i^th^* expert.

The *i^th^* parametric expert function, *f_i_*(***x****_n_*,*W_i_*) describes the relationship between the sEMG signals and the force applied at the fingertips. This function is restricted to be a linear vector such that $f_{i}\left({\text{x}}_{n},W_{i}\right)=W_{i}^{⊤}\left[{\text{x}}_{n}\;1\right]$ (1 represents a bias term) and *W_i_* is a $\left(d^{x}+1\right)\times d^{y}$ matrix corresponding to the weights associated with each sEMG signals and each DoF force signal. The probability distribution, $p\left({\text{y}}_{n}|{\text{x}}_{n},\theta_{i}^{e}\right)$, of the *i^th^* expert is taken to be a multivariate Gaussian distribution having mean *f_i_*(***x**_n_*,*W_i_*) and covariance $\chi_{i}^{-1}$, given by

(3) $$p\;\left({\text{y}}_{n}|{\text{x}}_{n},\theta_{i}^{e}\right)=N\;\left({\text{y}}_{n}|W_{i}^{\prime }\left[{\text{x}}_{n}\;1\right],\chi_{i}^{-1}\right)\;,$$

where $\theta_{i}^{e}=\left[W_{i},\chi_{i}\right]$; $\text{W}={\left\{W_{i}\right\}}_{i=1}^{M}$ is the multidimensional parameter weight matrix, and $\chi^{-1}={\left\{\chi_{i}^{-1}\right\}}_{i=1}^{M}$ is the covariance matrix.

**Figure 2:**
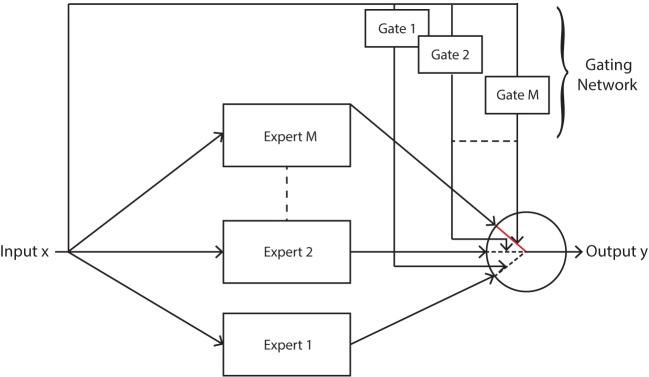
Mixture of experts model with soft competition, whereby only one expert is active at any given time. This expert is chosen probabilistically *via* the gating function.

The set of unknown model parameters for the model expressed in (1) is given by [**π**, ***Θ***] = [**π**, ***θ****^g^*, ***θ****^e^*]. Given that *N* training data points are available, then the joint likelihood of the finger force and sEMG signals is expressed as

(4) $$\begin{array}{ll} p\left(Y,X|\pi ,\Theta \right)= & \overset{N}{\underset{n=1}{\prod }}\;\overset{M}{\underset{i=1}{\sum }}\;\underset{\tilde{g_{i}}}{\underset{︸}{\pi_{i}N\;\left({\text{x}}_{n}|\mu_{i},\Lambda_{i}^{-1}\right)}}\\ & \times N\;\left({\text{y}}_{n}|W_{i}^{\prime }\left[{\text{x}}_{n}\;1\right],\chi_{i}^{-1}\right)\;, \end{array}$$

where $X=\left[{\text{x}}_{1},\ldots ,{\text{x}}_{N}\right]\in R^{N\times d^{x}}$, and $Y=\left[{\text{y}}_{1},\ldots ,{\text{y}}_{N}\right]\in R^{N\times d^{y}}$. The gating network ${\tilde{g}}_{i}$ is a Gaussian mixture model (GMM) and it divides the sEMG input space into separate Gaussian clusters. Hence, clustering (unsupervised classification) is performed using GMMs, and GMMs have been previously applied to hand movements as a means of performing classification with minimal computational complexity when compared to supervised learning classifiers (Chan and Englehart, 2003). The form of this gate results in singularities when maximum likelihood is used due to an ill-defined likelihood function (this causes a mixture component to collapse onto a single data point (Bishop, 2006)), hence further justifying the use of a Bayesian approach in this article. Expressing the likelihood as in equation (4) enforces soft competition between the experts such that only one expert will be dominant in a certain region of the input space (Jacobs et al., 1991), see Figure 2.

Discrete latent indicator variables, $Z={\left\{z_{\text{ni}}\right\}}_{i=1,n=1}^{M,N}\in R^{N\times M}$, are introduced such that if (***x****_n_*, ***y****_n_*) was generated from the *i^th^* expert then *z_ni_* = 1, else it is 0. Latent variables are introduced in order to simplify expression (4) since the sum over *M* experts is changed to a product over *M*. Thus the complete-data likelihood can be written as

(5) $$\begin{array}{ll} p\left(X,Y,Z|\pi ,\Theta \right)= & \overset{N}{\underset{n=1}{\prod }}\;\overset{M}{\underset{i=1}{\prod }}\left(\pi_{i}N\;\left({\text{x}}_{n}|\mu_{i},\Lambda_{i}^{-1}\right)\right.\\ & {\left.\times N\;\left({\text{y}}_{n}|W_{i}\prime \left[{\text{x}}_{n}\;1\right],\chi_{i}^{-1}\right)\right)}^{z_{\text{ni}}}, \end{array}$$

which is an equivalent representation of (4) incorporating an explicit latent variable.

#### 2.2.2 Priors

Conjugate priors are assigned to all the parameters except for the mixing coefficients **π** (which are treated as non-random variables). The priors used here follow on from the Bayesian literature.

The Gaussian-Wishart prior is used for the mean, ***μ***, and precision, **Λ**, of the gating GMM, given by

(6) $$\begin{array}{ll} p\left(\mu ,\Lambda \right)= & p\left(\mu |\Lambda \right)p\left(\Lambda \right)\\ = & \overset{M}{\underset{i=1}{\prod }}\;N\;\left(\mu_{i}|{\text{m}}_{0},{\left(\beta_{0}\Lambda_{i}\right)}^{-1}\right)\;W\left(\Lambda_{i}|B_{0},\nu_{0}\right), \end{array}$$

where *B*_0_ is a *d^x^* x *d^x^* symmetric, positive definite matrix, and *v*_0_ > *d^x^*–1 is the number of degrees of freedom of the Wishart distribution. Similarly, the prior distribution of the joint sEMG weights, ***W***, and precision matrix, ***χ***, for the linear regressor experts is given by a matrix-Normal-Wishart distribution expressed as

(7) $$\begin{array}{ll} p\left(\text{W},\chi |\text{a}\right)=\overset{M}{\underset{i=1}{\prod }}\; & MN\;\left(W_{i}|0,A_{i}^{-1},\chi_{i}^{-1}\right)\\ & \times W\left(\chi_{i}|Q_{0},\lambda_{0}\right), \end{array}$$

where *MN* is the matrix-Normal distribution. The matrix $A_{i}=\text{diag}\left(a_{i,1},\ldots ,a_{i,d^{x}+1}\right)$ and it is assigned the following hyperprior distribution

(8) $$\begin{array}{l} p\left(a_{i,j}\right)=Ga\left(a_{i,j}|c_{0},d_{0}\right). \end{array}$$

The parameter *A_i_* incorporates automatic relevance determination (ARD) since each input sEMG signal has an associated *a_i,j_* that forms part of the variance of the *i*th expert weights’ distribution (MacKay, 1995). ARD automatically imposes conditions such that if $a_{i,j}^{-1}=0$ then the corresponding sEMG signal ***x****^j^* will have little effect on the force.

The joint distribution of all the random variables can be expressed hierarchically as,

(9) $$\begin{array}{l} p\left(Y,X,Z,\mu ,\Lambda ,\text{W},\chi ,\text{a}|\pi \right)\\ =p\left(Y,X|Z,\pi ,\Theta \right)p\left(Z|\pi \right)p\left(\mu ,\Lambda \right)p\left(\text{W},\chi |\text{a}\right)p\left(\text{a}\right), \end{array}$$

where ***a*** = (***a***_1_,…,***a****_M_*). In order to simplify the assignment of hyperparameter values for the prior distributions, the extracted feature dataset was again standardized to zero mean and unit variance. This alleviates the issue of assigning hyperparameter values, removing the need to perform hyperparameter optimization. The hyperparameter values were set accordingly so as to define broad priors: ***m***_0_ is set using K-means, *β*_0_ = 0.01, *B*_0_ = *I*, and *v*_0_ = *d^x^* for the gates; and *Q*_0_ = *I*, λ_0_ = *d^y^*, *c*_0_ = 0.01, and *d*_0_ = 0.0001 for the experts.

### 2.3 Variational Bayesian Framework

Given the multivariate MoE and priors distributions for the random variables described in the previous sections, a learning algorithm is needed to train the model. An approximate Bayesian framework is desired in order to find the posterior distribution of the parameters *p*(***Θ,a***|*Y*). The marginal likelihood *p*(*Y*) (which appears in the denominator of Bayes’ theorem) is analytically intractable due to a multi-dimensional integral over the parameter space. The choice of conjugate-exponential distributions, along with a latent variable model is elegantly accommodated by the variational Bayes expectation-maximization (VBEM) framework. In short, the VBEM algorithm forms a lower bound of the marginal likelihood which is maximized iteratively so as to obtain a tight bound. Detailed information regarding the VBEM algorithm can be found in Beal (2003) and Beal and Ghahramani (2003).

Since conjugate priors for the model parameters were used, the functional form of the variational distributions will thus be the same as the priors. The optimal variational distributions are noted below, and expressed as q*(⋅). The VBM-step update equations are given in (10)-(16) while the VBE equations for the latent variables *Z* are shown in equatios (17)–(19). The equations given here are similar to the ones detailed in Baldacchino et al. (2016), however, some equations have been modified to cater for the multivariate output force signals.

The joint variational posterior distribution of the gates’ mean and covariance is a Gaussian-Wishart distribution, given by

(10) $$\begin{array}{l} q^{*}\left(\mu_{i},\Lambda_{i}\right)=N\;\left(\mu_{i}|{\text{m}}_{i},{\left(\beta_{i}\Lambda_{i}\right)}^{-1}\right)\;W\left(\Lambda_{i}|B_{i},\nu_{i}\right), \end{array}$$

where

(11) $$\begin{array}{ll} {\text{m}}_{i} & =\frac{\beta_{0}{\text{m}}_{0}+\sum_{n=1}^{N}\;r_{\text{ni}}{\text{x}}_{n}}{\beta_{i}}\;,\;\beta_{i}=\beta_{0}+N_{i},\\ B_{i}^{-1} & =B_{0}^{-1}+\overset{N}{\underset{n=1}{\sum }}\;r_{\text{ni}}{\text{x}}_{n}{\text{x}}_{n}^{\prime }+\beta_{0}{\text{m}}_{0}{\text{m}}_{0}^{\prime }-\beta_{i}{\text{m}}_{i}{\text{m}}_{i}^{\prime }\\ \nu_{i} & =\nu_{0}+N_{i},\;N_{i}=\overset{N}{\underset{n=1}{\sum }}\;r_{\text{ni}},\;r_{\text{ni}}=E\left[z_{\text{ni}}\right]. \end{array}$$

The joint variational posterior distribution of the expert functions’ mean and covariance is a matrix-Normal-Wishart distribution having the following form

(12) $$\begin{array}{l} q^{*}\left(W_{i},\chi_{i}\right)=MN\;\left(W_{i}|{Ŵ}_{i},\chi_{i}^{-1},L_{i}\right)\;W\left(\chi_{i}|\lambda_{i},Q_{i}\right), \end{array}$$

where

(13) $$\begin{array}{l} {Ŵ}_{i}=L_{i}\left[X\;{\text{1}}_{N\times 1}\right]\prime V_{i}Y\\ L_{i}={\left(\left[X\;{\text{1}}_{N\times 1}\right]\prime V_{i}\left[X\;{\text{1}}_{N\times 1}\right]+{ϒ}_{i}\right)}^{-1}\\ V_{i}=\text{diag}\left(E\left[z_{1i}\right],\ldots ,E\left[z_{\text{ni}}\right]\right)\\ \lambda_{i}=\lambda_{0}+N_{i}\\ Q_{i}^{-1}=Q_{0}^{-1}+Y\prime V_{i}Y-{Ŵ}_{i}^{\prime }\left(X\prime V_{i}X+{ϒ}_{i}\right){Ŵ}_{i}. \end{array}$$

The term ϒ*_i_* = ℰ[*A_i_*] is defined in equation (16). The variational distribution for the ARD parameters is

(14) $$\begin{array}{l} q^{*}\left(a_{i,j}\right)=Ga\left(a_{i,j}|c_{i},d_{i,j}\right), \end{array}$$

where

(15) $$\begin{array}{l} c_{i}=c_{0}+0.5d^{y},\;d_{i,j}=d_{0}+0.5\xi_{i,j}\\ \xi_{i,j}=d^{y}{\left(L_{i}\right)}_{j,j}+\lambda_{i}{\hat{\text{w}}}_{i,j}Q_{i}{\hat{\text{w}}\prime }_{i,j}, \end{array}$$

where (*L_i_*)*_j,j_* is the *j*th diagonal element of *L_i_*, and ${\hat{\text{w}}}_{i,j}\in R^{1\times d^{y}}$ is the *j*th row of Ŵ_i_. Using the statistic of a mean from a Gamma distribution, then

(16) $$\begin{array}{l} {ϒ}_{i}=E\left[A_{i}\right]=\text{diag}\;\left(\frac{c_{i}}{d_{i,1}},\ldots ,\frac{c_{i}}{d_{i,d^{x}+1}}\right)\;. \end{array}$$

The VBE-step consists of updating the variational distribution of *Z* and the relevant equations are listed below. The variational distribution for the latent indicator variables follows a multinomial distribution, such that

(17) $$\begin{array}{ll} \text{ln}\;q^{*}\left(Z\right) & =\overset{N}{\underset{n=1}{\sum }}\;\overset{M}{\underset{i=1}{\sum }}\;z_{\text{ni}}\;\text{ln}\;r_{\text{ni}}\;\text{and}\;r_{\text{ni}}=\frac{\gamma_{\text{ni}}}{\sum_{l=1}^{M}\;\gamma_{\text{nl}}}, \end{array}$$

(18) $$\begin{array}{ll} \gamma_{\text{ni}} & =\pi_{i}\text{exp}\;\left\{\frac{1}{2}\;\left(\;\text{ln}\;{\hat{\Lambda }}_{i}+\;\text{ln}\;{\hat{\chi }}_{i}-\varpi_{\text{ni}}-\xi_{\text{ni}}\right)\;\right\}\;. \end{array}$$

The terms required in equation (18) are,

(19) $$\begin{array}{ll} \text{ln}{\tilde{\Lambda }}_{i} & =E\left[\text{ln}|\Lambda_{i}|\right]\\ & =\overset{d^{x}}{\underset{j=1}{\sum }}\psi \;\left(\frac{\nu_{i}+1-j}{2}\right)+d^{x}\;\text{ln}2+\text{ln}|B_{i}|,\\ \text{ln}{\tilde{\chi }}_{i} & =E\left[\text{ln}|\chi_{i}|\right]\\ & =\overset{d^{y}}{\underset{j=1}{\sum }}\psi \left(\frac{\lambda_{i}+1-j}{2}\right)+d^{y}\;\text{ln}2+\text{ln}|Q_{i}|, \end{array}$$

$$\begin{array}{ll} \varpi_{\text{ni}} & =\text{Tr}\left(E\left[\Lambda_{i}\right]E\left[\left({\text{x}}_{n}-\mu_{i}\right)\left({\text{x}}_{n}-\mu_{i}\right)\prime \right]\right)\\ & =\nu_{i}\left({\text{x}}_{n}-{\text{m}}_{i}\right)B_{i}\left({\text{x}}_{n}-{\text{m}}_{i}\right)\prime +\beta_{i}^{-1}d^{x},\\ \xi_{\text{ni}} & =\text{Tr}\left(E\left[\chi_{i}\right]E\left[\left({\text{y}}_{n}-\left[{\text{x}}_{n}\;1\right]W_{i}\right)\left({\text{y}}_{n}-\left[{\text{x}}_{n}\;1\right]W_{i}\right)\prime \right]\right)\\ & =\lambda_{i}\left({\text{y}}_{n}-\left[{\text{x}}_{n}\;1\right]{Ŵ}_{i}\right)Q_{i}\left({\text{y}}_{n}-\left[{\text{x}}_{n}\;1\right]{Ŵ}_{i}\right)\prime \\ & \;\;+d^{y}\left[{\text{x}}_{n}\;1\right]\prime L_{i}\left[{\text{x}}_{n}\;1\right]. \end{array}$$

where *ψ*(⋅) is the digamma function.

The update equation for the mixing coefficients is obtained by maximizing the variational lower bound with respect to the mixing coefficients. The update equation is given by

(20) $$\begin{array}{l} \pi_{i}=\frac{1}{N}\overset{N}{\underset{n=1}{\sum }}\;r_{\text{ni}}. \end{array}$$

The equation given above was used to determine the number of mixtures in GMMs (Corduneanu and Bishop, 2001), and it was later extended to a MoE framework in Baldacchino et al. (2016). Using (20), any surplus experts will have their π_i_ driven to zero, effectively removing them from the model. This equation needs to be interleaved into the iterative procedure since (18) depends on **π**. Following on from previous authors the mixing coefficients are updated after a pass of the variational posterior distribution update equations. The equations presented in this section are summarized in Algorithm 1.

**Algorithm 1:** Multivariate VBEM MoE.

**Initialize the hyperparameters**:
- GMM gates, ***m***_0_, *β*_0_, ***B***_0_ and *ν*_0_,
- Linear experts *ρ*_0_, λ_0_, *c*_0_, *d*_0_ and ${ϒ}_{\text{i}}^{\left(\text{0}\right)}=\frac{{\text{c}}_{\text{0}}}{{\text{d}}_{\text{0}}}{\text{I}}_{{\text{d}}_{\text{x}}}$ ∀ *i*.
- Initialize $\gamma_{\text{ni}}^{\left(\text{0}\right)}∼U\left[\text{0},\text{1}\right]$ ∀*i*, *n*.
**for** *k* = 0: stopping criteria
**VBM-step:**
- Evaluate mixing coefficients **π**^(^*^k^*^+1)^ using (20)
- Update the gate parameters using (11).
- Update the expert parameters using (13), (15) and (16).
**VBE-step:**
- Update for latent variables *Z* using (17)–(19).
**end for**

### 2.4 Posterior Predictive Distribution

The posterior predictive distribution allows one to perform predictions of the output force signals to new unseen sEMG signals. This distribution also gives information regarding the variance of the predictions, and thus confidence bounds can be calculated. The posterior predictive distribution is expressed as $p\left(y_{n\prime }|{\text{x}}_{n\prime },D\right)$, where 𝒟 = [*Y*, *X*], and n′=N+1 is the new data point. For the mixture of experts described in this article, the predictive distribution is given by,

(21) $$\begin{array}{l} p\left({\text{y}}_{n\prime }|{\text{x}}_{n\prime },D\right)=\overset{M}{\underset{i=1}{\sum }}\;\Omega_{i,n\prime }T\;\left({\text{y}}_{n\prime }\left|\left[{\text{x}}_{n\prime }\;1\right]{Ŵ}_{i},\kappa_{i}\Sigma_{i},\kappa_{i}\;\right.\right) \end{array}$$

where ${\left\{\Omega_{i,n\prime }\right\}}_{i=1}^{M}$ take value 1 with probabilities ${\left\{g_{i,n\prime }^{\text{MAP}}\right\}}_{i=1}^{M}$, respectively. $g_{i,n\prime }^{\text{MAP}}=g_{i}\left({\text{x}}_{n\prime },\pi_{i},\theta_{i\text{MAP}}^{g}\right)$ using (2) at the maximum *a posteriori* (MAP) estimates $\theta_{\text{MAP}}^{g}=\left\{\mu_{\text{MAP},}\Lambda_{\text{MAP}}\right\}$ obtained from the posterior distribution (10), and the final value for **π**. At any given time *n*′ only one ${\left\{\Omega_{i,n\prime }\right\}}_{i=1}^{M}$ can be 1 (the rest are zero) corresponding to the gate with the largest probability. $T\left(\left[{\text{x}}_{n\prime }\;1\right]{Ŵ}_{i},\kappa_{i}\Sigma_{i},\kappa_{i}\right)$ is a multivariate Student-*t* distribution with *κ*_i_ = λ*_i_* − *d^y^* + 1 degrees of freedom, mean $\left[{\text{x}}_{n\prime }\;1\right]{Ŵ}_{i}$ and scale matrix *κ*_i_Σ*_i_*, where $\Sigma_{i}=Q_{i}{\left(1+\left[{\text{x}}_{n\prime }\;1\right]\prime L_{i}\left[{\text{x}}_{n\prime }\;1\right]\right)}^{-1}$. The relevant statistics for prediction are

(22) $$\begin{array}{ll} E\left[{\text{y}}_{n\prime }\right] & =\overset{M}{\underset{i=1}{\sum }}\;\Omega_{i,n\prime }\left[{\text{x}}_{n\prime }\;1\right]{Ŵ}_{i},\\ \text{cov}\left[{\text{y}}_{n\prime }\right] & =\overset{M}{\underset{i=1}{\sum }}\;\Omega_{i,n\prime }\frac{\Sigma_{i}^{-1}}{\kappa_{i}-2}. \end{array}$$

### 2.5 Implementation

The multivariate VBEM MoE algorithm, given in Algorithm 1, was trained on the sEMG/force data from all 40 subjects, and all the 6 force signals were trained together. The gate divisions on the input space are common to all the outputs. Since 9 movements and a rest period between repetitions and movements are considered here, the algorithm is initialized with 10 experts. The algorithm was run 100 times with random initialization for ***γ*** and ***m***_0_ so as to avoid the local maxima problem. The model with the largest lower bound was chosen as being the best model to represent the data. The algorithm was run for all the FE representations listed in Table 3, and a comparison between them is considered in Section 3.

### 2.6 Sensitivity Analysis

In this article, the authors apply SA techniques with the intention of assessing the importance of the number of electrodes to the performance of the modeling framework. Interested readers are referred to Saltelli et al. (2000, 2008) for an in-depth exploration of SA. Here, the regression-based global SA technique developed in Xu and Gertner (2008) is applied since this method accommodates correlated input signals. The contribution of an individual sEMG signal to the variance of the model force output, *V_j_*, is decomposed into two parts: the uncorrelated contribution *VU_j_*, which describes variations unique to a sEMG input ***x**^j^* which are independent of the other sEMG inputs, and correlated contribution *VC_j_*, which explains variations of an sEMG input ***x****^j^* which are correlated with other sEMG signals (Xu and Gertner, 2008)

(23) $$\begin{array}{l} V_{j}=VU_{j}+VC_{j}. \end{array}$$

This discrimination between uncorrelated and correlated contributions highlights, respectively, if the sEMG input itself dominates or if the correlated variations among the sEMG signals dominate. The three indices found in equation (23) can be calculated using the regression-based SA equations found in Xu and Gertner (2008). The final indices of interest are the first-order sensitivity indices given by the total *S_j_*, uncorrelated *SU_j_* and correlated *SC_j_* contributions of sEMG input ***x****^j^* such that

(24) $$\begin{array}{l} S_{j}=\frac{V_{j}}{V},\;SU_{j}=\frac{VU_{j}}{V},\;SC_{j}=\frac{VC_{j}}{V}, \end{array}$$

where $V=\frac{1}{N_{\mathrm{lhs}}}\sum_{n}\;{\left({\text{y}}_{n}-\bar{\text{y}}\right)}^{2}$ is the estimated variance of the force output, and ***y***¯ is the mean of the force signal. A Latin hypercube sampling (LHS) procedure is used to generate *N_lhs_* rank-correlated samples for *X* from which the output ${\left\{\text{y}\right\}}_{n=1}^{N_{\mathrm{lhs}}}$ can be generated using the final MoE model. The LHS strategy used here follows that given in Iman and Conover (1982) since it takes into account the correlations between the inputs when generating samples. Using the sensitivity indices given in equation (24), examination of spurious sEMG signals is possible since they will affect the force output only due to their strong correlations with other significant sEMG inputs (that is, they will have a very low *SU_j_* compared to *SC_j_*). The regression-based SA approach relies on an approximate linear relationship between the sEMG signals and the force. This is not a bad assumption for the data used in this article since in Gijsberts et al. (2014), an average *R*^2^ = 60% was achieved for a linear model using RMS FE.

## 3 Results

The multivariate Bayesian MoE algorithm for simultaneous regression/classification was applied to the problem of predicting finger force dynamics and classifying finger movements from the Ninapro dataset. The results are reported here with a comparative evaluation against previous results (Gijsberts et al., 2014): firstly the results of finger force regression are presented and secondly classification. Lastly, SA is performed in order to assess the importance of the electrodes’ contribution to the output force signals.

### 3.1 Finger Force Regression Using Test Data

The performance of the final regression models is analyzed using the normalized root mean square error (NRMSE) measure, given by

(25) $$\begin{array}{l} \mathrm{NRMSE}=\frac{\sqrt{\left(1∕N\right)\sum_{n=1}^{N}\;{\left({\text{y}}_{n}-{\hat{\text{y}}}_{n}\right)}^{2}}}{{\text{y}}^{\max}-{\text{y}}^{\min}}, \end{array}$$

and the coefficient of determination (*R*^2^), given by

(26) $$\begin{array}{l} R^{2}=1-\frac{\sum_{n}{\left({\text{y}}_{n}-{\hat{\text{y}}}_{n}\right)}^{2}}{\sum_{n}{\left({\text{y}}_{n}-\bar{\text{y}}\right)}^{2}}. \end{array}$$

where ${\hat{\text{y}}}_{n}$ is the model predicted output at time instance *n*, and $\bar{\text{y}}$ is the mean of ***y*** These two statistics were chosen because of their wide use by the bio-robotics and rehabilitation/prosthetics communities in assessing the quality of their models.

**Figure 3:**
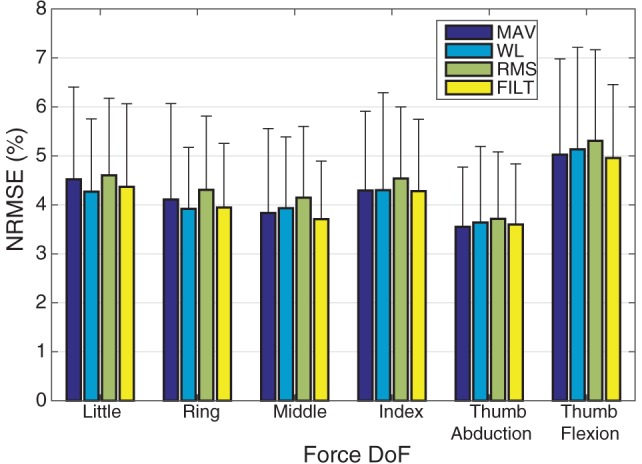
A plot of NRMSE averaged over the 40 subjects for the 6 DoF. The error bars indicate unit SD.

The average normalized root mean square error (NRMSE) for each force DoF across all subjects, is plotted for all the different FEs in Figure 3. All FEs perform similarly and low NRMSE values are recorded. The multivariate VBEM MoE algorithm performs consistently well across all subjects since the SDs associated with each DoF are small compared to their mean values (i.e., the coefficients of variation are overall less than 30%). Focusing on the FILT FE as being fairly typical, the best results are obtained for the thumb abduction force signal (3.6 ± 1.2%), while the worst result is for the thumb flexion force signal (4.96 ± 1.5%). Prediction of little, ring, middle and index flexion force signals have similar performance.

**Figure 4:**
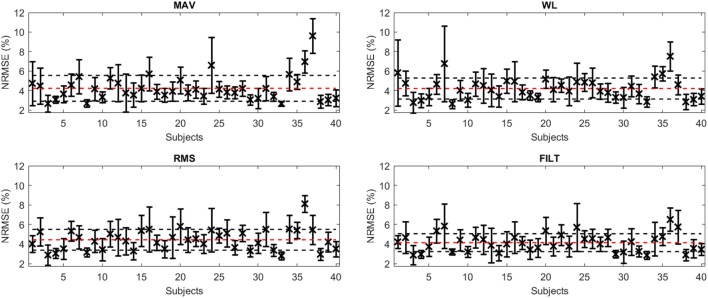
A plot of average NRMSE for the model obtained for each subject for all FEs (x), along with ±1 SD (black error bars). The red dashed line represents the mean NRMSE, while the horizontal black dashed lines represent ±1 SD from this mean.

Figure 4 shows the average NRMSE for all subjects (averaged over the 6 DoF force activations) for each FE, including unit SD (given by the black vertical lines). The performance of the algorithm is fairly consistent across all subjects, with most subjects achieving a level of NRMSE below 5%. Subject 37 for MAV has the worst overall NRMSE of 9.61 ± 1.76% while Subject 36 performs the worst for the other FEs. The best results were achieved by Subject 8 for WL and Subject 33 for MAV having an NRMSE of 2.64 ± 0.46 and 2.64 ± 0.22%, respectively (Subject 33 has the worst NRMSE for RMS and FILT). The mean (over all DoF and all subjects) and corresponding unit SD for both NRMSE (given by red and black horizontal lines in Figure 4) and *R*^2^ are reported in Table 4. Overall, the FILT FE has the best performance with the other FEs having very similar performance.

**Table 4:** Average *R*^2^ and NRMSE including 1 SD.

Feature type	Average *R^2^*	Average NRMSE
MAV	90.54 ± 6.93%	4.22 ± 1.33%
WL	90.75 ± 5.07%	4.20 ± 1.08%
RMS	89.81 ± 5.00%	4.44 ± 1.06%
FILT	90.91 ± 4.50%	4.14 ± 0.92%

**Figure 5:**
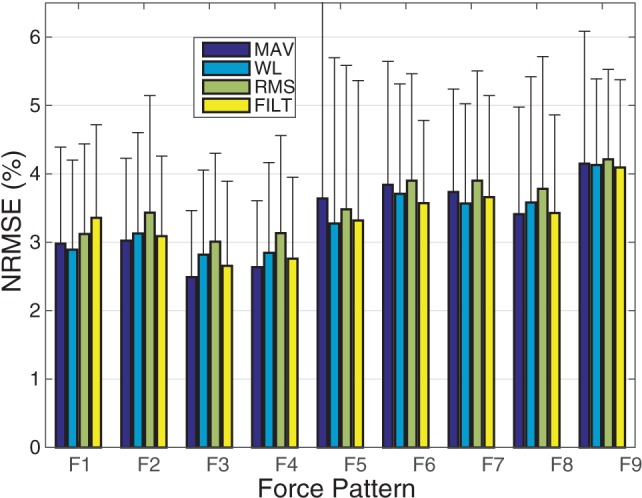
A plot of the average NRMSE for each individual force pattern. The error bars indicate unit SD.

To investigate the performance of the models on each of the 9 force patterns, the average NRMSE per pattern is shown in Figure 5. Similar to that reported in Gijsberts et al. (2014), patterns involving the individual activation of the four fingers (F1-F4) are all characterized by slightly better performance overall (i.e., by NRMSE values around 3%). The remaining movements (F5–F9) have a slightly worse performance (between 3.5 and 5%), especially those movements involving flexion of the thumb (F6 and F9); this is consistent with the results shown in Figure 3, where thumb flexion shows the worst NRMSE result.

#### 3.1.1 Results for Subject 1

The results reported in this section concern Subject 1 (so as to compare to Gijsberts et al. (2014)) and the model obtained with the RMS FE is used since this model has the lowest NRMSE for this particular subject.

**Figure 6:**
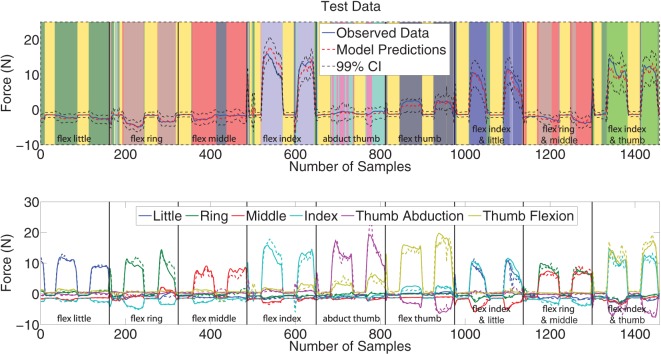
Top plot: Predictions (red) versus observed data (blue) including 99% confidence intervals (dashed black) on the test data of the fourth DoF force activation. The background of colors represents the individual experts, and black vertical line indicates when a movement starts. Bottom plot: A plot of observed (solid) and predicted (dashed) forces for the second and fifth repetitions of all nine force patterns. Each box represents a force pattern, and each color corresponds to a force measurement.

The top plot of Figure 6 shows the predictions (red) obtained on the test data (blue) for the fourth force DoF (index flexion). The signals are plotted on a background of colors where each color corresponds to a particular expert. The black dashed lines represent the 99*%* confidence intervals which are calculated using (22) hence arising naturally from the Bayesian inference framework. The confidence intervals visibly enclose most of the observed data (blue). The model predictions of the force closely follow the measured data indicating that the multivariate MoE model is a suitable model for performing force regression from sEMG signals; at least so far as Subject 1 is concerned.

An equally interesting result is the assignment of experts to the data points. The different movements are immediately identified as being separate due to being allocated a different expert (denoted by a different color in Figure 6). The experts allotted to the different movements in the training set are also the same ones used in the test set for corresponding movements. Details of expert assignment are discussed further in Section 3.2.

The predictions for all 6 DOF force measurements on the test data is shown in the bottom plot of Figure 6. All the force measurement signals and force patterns are predicted well, with the predicted and measured data visibly following the same overall trends. The model even learned the involuntary negative forces, which was also observed by Gijsberts et al. (2014) and they attribute these forces to synergistic or compensatory mechanisms.

### 3.2 Finger Movement Classification

Classification can be seen as a natural by-product of the VBEM MoE algorithm presented in this article. The learning algorithm consists of soft competition among the experts, such that only one expert dominates in different regions of the input space. The dominant expert is probabilistically chosen by assessing the gates using (2).

**Figure 7:**
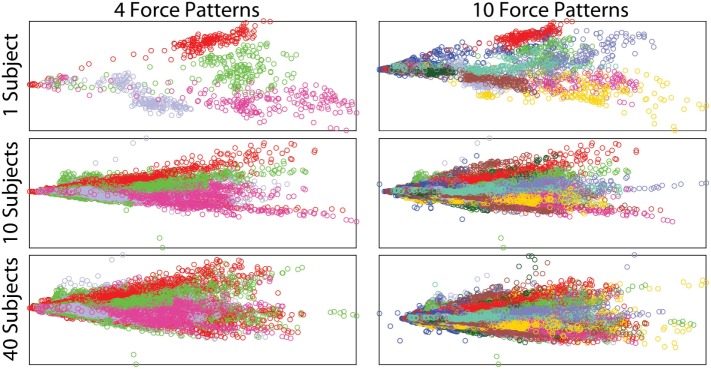
Two principal components of the filtered sEMG signals, showing the effects of increasing the number of subjects and force patterns. Each color represents a different force pattern.

Insight into the relation between the different force movements and the sEMG signals is obtained *via* principal component analysis (PCA). Using PCA on the sEMG signals, the first two principal components can be plotted against each other, with the different force patterns each taking on a unique color, as shown in Figure 7. In this plot, the columns indicate 4 and 10 (9 movements plus rest) force patterns, respectively, while the rows show the effect of increasing the number of subjects. Even when just one subject is considered, increasing the number of movements results in the force patterns having lots of overlap, hence making it harder to distinguish between the different movements. Similarly, increasing the number of subjects also results in a large overlap. This figure also highlights the high variability in the recorded sEMG signals between subjects, and it is expected that this variability will be reflected in large deviations in classification performance. High variability among subjects was also observed by Atzori et al. (2012) for different hand movements.

**Figure 8:**
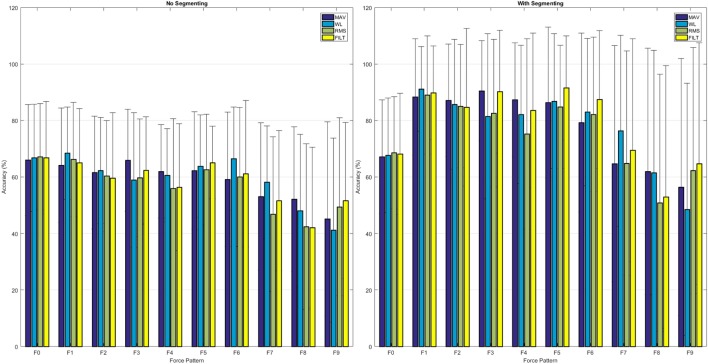
Average classification accuracy for each individual force pattern, including rest, for each of the FEs: left, using the continuous data, right, using the segmented data.

**Figure 9:**
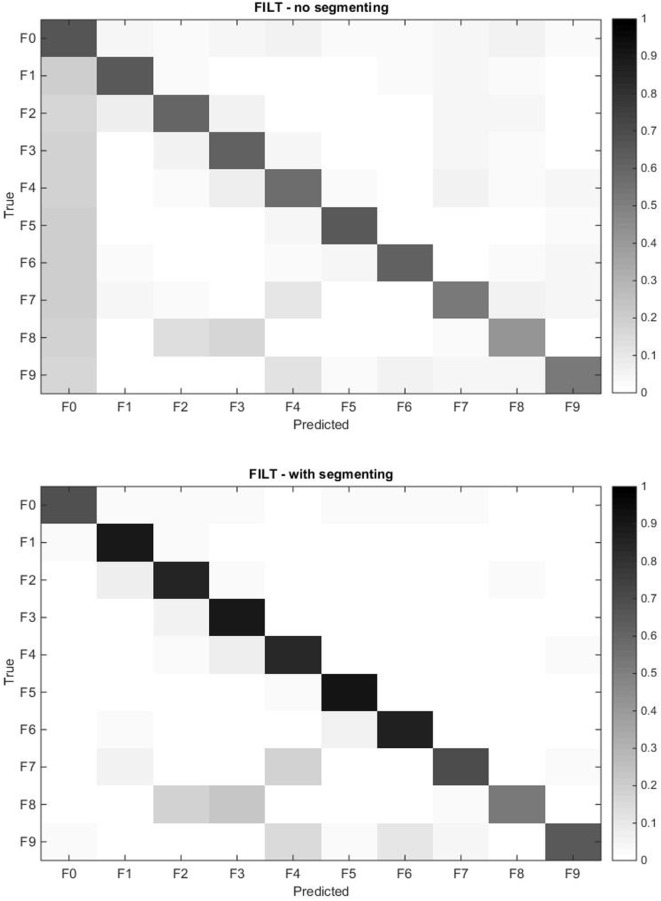
Confusion matrix for the models obtained with the FILT FE averaged over all 40 subjects for all force patterns including rest. The rows represent the true class, and each cell corresponds to the prediction accuracy of each class. The top plot corresponds to the continuous data, while the bottom plot corresponds to the segmented data.

A common metric for assessing the quality of the classifier is the classification accuracy, which is the proportion of correctly classified instances to the total number of instances. The left-hand plot in Figure 8 shows the average classification accuracy for each force pattern where F0 is the rest position, for all the FEs. All FEs achieve similar performance, and multifinger movements have a lower accuracy; more information can be obtained by analyzing the confusion matrix given in the top plot of Figure 9.

The confusion matrix profiles the misclassification of movements since correct predictions would result in a distinct black diagonal (left to right), while any non-clear off-diagonal cells are indicative of misclassifications. The top plot in Figure 9 shows that the VBEM MoE framework is consistent in correctly classifying movements, since the diagonal is prominent. The most conspicuous misclassification is given by the non-clear first column indicating that all force patterns are sometimes mistaken as rest, that is, the absence of movement. This phenomenon was also observed by Kuzborskij et al. (2012), and they attribute this to: 1/the data contains rest-to-movement segments which causes ambiguity and these segments are technically neither rest nor non-rest, and 2/when windowing is used, some windows include sections of rest and non-rest samples and so these samples are again neither rest nor non-rest. For the FILT FE only point 1 is valid (since no windowing is used), and this is enough to confuse the algorithm into thinking that these regions of the data are associated with no movement. In Chan and Englehart (2003), it was reported that this type of error is treated as being acceptable since it is expected that due to mechanical inertia, a prosthesis would not be able to respond to transitory misclassifications. In the literature regarding finger movements classification, the rest position is either ignored or the sEMG data stream is segmented in such a way that regions of ambiguity are removed from the training set.

The right hand plot of Figure 8 and the bottom plot of Figure 9 show the results when only sections of the data set are considered when calculating classification accuracy, that is, each movement is divided into three equally sized segments and only labels from the center segment were retained (no retraining was performed). A higher accuracy is now attainable: single finger movements and multi-finger movements have an average accuracy of 87.89% and 62.39%, respectively.

Referring back to the confusion matrix plots (Figure 9) gives useful insight into why multi-finger movement achieves lower accuracy than single finger movement. Movement F8 (flexion of ring and middle fingers) has the lowest classification accuracy from all movements. The algorithm mostly misclassified F8 for the corresponding individual finger movements of F2 (flex ring) and F3 (flex middle). The same trend is observed with movements F7 (flexion of index and little fingers) and F9 (flexion of index finger and thumb).

### 3.3 Reducing the Number of Electrodes *via* SA

The SA procedure outlined in Section 2.6 is applied to the dataset using the final MoE model obtained with the FILT FE (similar results were obtained using the other models, so only the results of one FE are shown here). All 12 sEMG signals are treated as individual inputs, and the SA results revealed that the contributions of all the sEMG input signals to the variance of the force outputs are mainly due to the correlations with other inputs, since all inputs had a low *SU* index compared to *SC* index.

**Figure 10:**
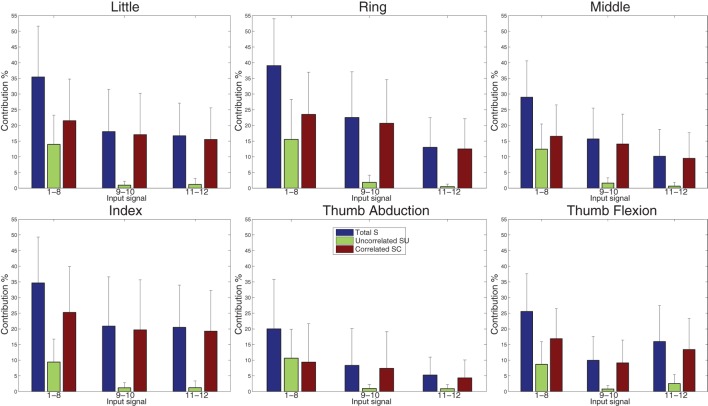
Variance contribution for the six force signals using regression-based sensitivity analysis for correlated inputs (using the MoE model obtained with FILT FE) for grouped sEMG input signals, averaged over all 40 subjects. The large SU values (green) indicate that the group of inputs 1–8 is influential.

The SA is performed again but this time the sEMG input signals are grouped according to their electrode location. Thus the groups comprise of electrodes 1–8 (forearm), 9–10 (finger extensor and flexor), and 10–11 (biceps and triceps)—refer to Figure 1. The three sensitivity indices, averaged over all 40 subjects, for all the output force signals for these groups of inputs are shown in Figure 10. The *SU* index for electrodes 1–8 is now significant (and comparable to the *SC* index) for most outputs. Hence, the sEMG electrodes located around the forearm are necessary for describing the relationship between force at the fingertips and the sEMG signals because this group of inputs’ individual contribution is important. The other 2 groups of inputs are almost entirely dominated by their correlated effect, and so their individual contribution is low.

**Figure 11:**
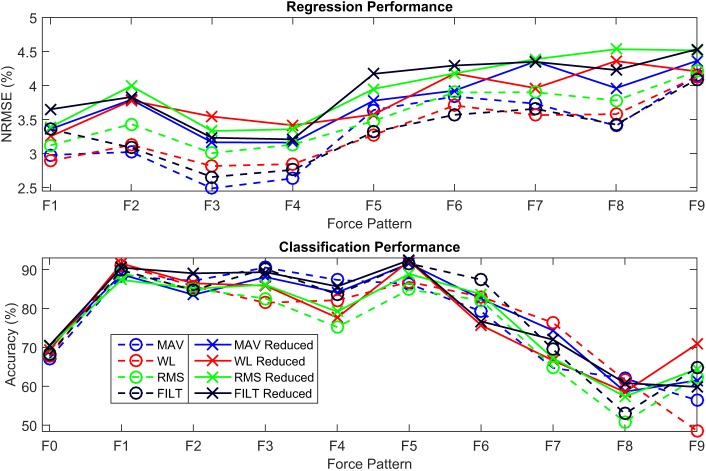
Comparison between the models obtained using all the electrodes (dashed) and using the 8 electrodes around the forearm only (solid) for 4 FEs (MAV, WL, RMS, and FILT). Top: Average NRMSE for each force pattern for assessing the quality of the force regression. Bottom: Average classification accuracy for each force pattern, including rest (F0), for assessing the quality of classification.

The multivariate VBEM MoE algorithm was run again but this time only the 8 electrodes around the forearm were used as inputs to the model. The plot shown in Figure 11 shows the performance of the models on the reduced input set (solid lines) versus that of the full input set (dashed lines) for both regression and classification over the different force patterns. The top plot shows the quality of the models for the force regression given by the average NRMSE for the different force activations considered. A slight degradation in NRMSE is observed for the reduced input set (across all FEs), however, the models are still performing exceptionally well. The bottom plot demonstrates the quality of classifying the different finger movements including the rest position using the average classification accuracy. In this case, the reduced set and full set models have similar performance, with no clear winner.

## 4 Discussion

### 4.1 Regression

The results and observations obtained for the force regression using the multivariate VBEM MoE are comparable to those reported in the literature, for example (Castellini and Kõiva, 2012; Gijsberts et al., 2014). Predictions of thumb movements are worse than for the other fingers due to no sEMG activity being recorded from the majority of the thumb muscles since these are located at the wrist (and hence are not usually available on the amputee’s stump) (Kõiva et al., 2012; Gijsberts et al., 2014).

In the benchmark set by Gijsberts et al. (2014), overall *R*^2^ values of 91.74 and 88.93% for marginal discrete wavelet transform (mDWT) and RMS, respectively, for a exp-χ^2^ kernel ridge regression were reported. The results of Table 4 indicate that the VBEM MoE algorithm achieves similar performance to that given in Gijsberts et al. (2014) but with the use of a much simpler FE representation for sEMG signals. The mDWT has a considerably higher feature dimensionality (36 inputs) than the FEs considered in this article (12 inputs). The use of a low dimensional FE allows for faster feature computation of the windowed sEMG signals compared to the mDWT in the preprocessing stage. Faster training times are also achieved when a smaller input dimension is used in any algorithm. It has also been shown that TD features performed better than frequency domain and WTs in real-time applications (Englehart and Hudgins, 2003).

The NRMSE values for each finger force pattern are comparable to those reported in the literature, such as (Castellini and Kõiva, 2012; Gijsberts et al., 2014). The *R*^2^ values are not reported for the individual force patterns since the sum of error over each force pattern is not necessarily 0, and so the *R*^2^ value can go negative although the model provides a good fit. Therefore, it is not possible to perform a direct comparison to the *R*^2^ values reported in Gijsberts et al. (2014) since it would result in an erroneous interpretation. However, the NRMSE values reported here highlight what the authors in Gijsberts et al. (2014) and others have established: force signals from single finger movements are easier to predict than from multiple finger movements and thumb movements.

### 4.2 Classification

The misclassification of multiple finger movements reported in this article tends to be represented by the corresponding individual finger movements. This result seems to suggest that multiple finger movements can be accurately represented by their corresponding individual finger movements implying that the sEMG signals of the multiple finger movements can be decomposed into the individual sEMG signals. This outcome appears to give weight to the hypothesis presented in Castellini and Kõiva (2012) whereby the authors suggested that for example flexion of ring + middle would be statistically similar to flexion of little + ring + middle. To the best of the authors’ knowledge, this phenomenon of sEMG signals of some multiple finger movements being similar to the sEMG signals of the individual fingers has not been analyzed in the literature. Possible explanations for this could be that similar finger movements have not been tackled (some articles do not specify which multiple finger movements are being analyzed), and confusion matrices are not reported hence by-passing the opportunity to assess in detail how the classifier is misclassifying movements.

The classification accuracy reported in this article is similar to that found in the literature, refer to Table 1. The algorithm presented here is capable of classifying finger movements which is the result of a by-product from regression analysis—no extra computational expense is incurred. Another important point to note is that this is the first instance of simultaneous regression and classification of finger movements *via* sEMG signals using MoE models.

### 4.3 SA

The initial SA investigation indicated that the contribution to the force variance was a result of correlated contributions of all the sEMG signals. This result seems to suggest that the VBEM MoE learning algorithm is not sensitive to the nominal placement of electrodes since the uncorrelated contribution of all the electrodes was low suggesting that individual contribution of the electrodes themselves are not important. This result supports the research performed in Hargrove et al. (2008), where the authors report that the pattern recognition framework is insensitive to nominal electrode placements, however, it is sensitive to electrode displacement during the training/testing phase. Hence, the same locations need to be used for training and testing in order for a classifier/regressor to have a high accuracy.

Good force regression and movement classification was achieved by the VBEM MoE model on a reduced input set which consists of a dense sampling of electrodes around the forearm. This setup in which no relevant muscles are targeted was considered by Castellini and Kõiva (2012) such that 9 electrodes were uniformly positioned around the forearm. The regression results obtained here are comparable to those reported by Castellini and Kõiva (2012). Reducing the number of electrodes reduces the overall cost in terms of both physical cost (less electrodes) and computational cost (training the model). With a training dataset of around 3,000 samples, an average training time of 114 s for the full input set and an average of 93 s for the reduced input set was achieved. Therefore, SA techniques provide a structured methodology for reducing the number of electrodes needed for clinical application.

## 5 Conclusion

Following on from preliminary work, this article provides an in-depth analysis of the use of multivariate Bayesian mixture of experts models using sEMG signals for refined control of prosthetics with multiple DoF. Bayesian inference is a novel concept within the sEMG community and it allows uncertainties to be naturally incorporated into the model structure, supporting a fuller description of the model. The use of a MoE model provides simultaneous finger force regression and finger movement classification at no extra computational cost. The MoE model favors a more natural interpretation between the sEMG and force signals by automatically separating out the data into individual finger movements. This feature relates the model with the underlying biological and physical properties of the data. The MoE model is mathematically and (potentially) computationally demanding compared to other regression and classification methods; however, this is balanced by the extra capability that is obtained.

The method described in this work achieves high performance using low-dimensional feature extraction techniques for the sEMG signal. Accurate force predictions and movement classification were obtained for several finger movements, across all 6 DoF force activations and all 40 subjects. Examination into the effects of a reduced set of sEMG inputs using SA techniques enabled a structured investigation into the influence of the sEMG inputs on the force regression/movement classification performance. It was concluded that the dense sampling of electrodes around the forearm had the greatest influence on the output force DoF. Retraining the models on a reduced input set resulted in similar performance for both the force regression and movement classification when compared to the full input set models.

The next phase of research is to apply this framework to finger force data collected from transradial amputees, and compare performance to that obtained by healthy subjects. It is foreseen that the algorithm will achieve high accuracy, thus providing an exciting novel method of controlling state-of-the-art dexterous myoprosthetics.

## Author Contributions

TB ran simulations and wrote up most of the article. WJ and SA were involved with the literature review and introduced TB to the NinaPro database. WJ, SA, KW, and JR were involved in discussions regarding machine learning algorithm. JR was PI.

## Conflict of Interest Statement

The authors declare that the research was conducted in the absence of any commercial or financial relationships that could be construed as a potential conflict of interest.
